# Medical Opinion and Movement

**Published:** 1909-10-23

**Authors:** 


### Medical Opinion and Movement.

At the recent French Surgical Congress held at Paris an interesting discussion took place on the ante- and post-operative treatment in abdominal surgery. The disinfection of the skin, Murphy's treatment with continuous rectal irrigation with normal saline, methods of drainage, and many other points came under discussion, but the question that raised most comment was the new method of treatment advocated by one or two leading spirits, namely, the so-called *lever précoce*, or getting the patient up on the third or fourth day after operation. The treatment was almost universally condemned as rash and inexpedient. The chief danger is, of course, thrombosis and embolism. Dr. Villar recorded the fact that a surgeon of his acquaintance who conceived the idea that rest in bed actually predisposed to embolism, allowed his patients up soon after operation in order to avoid this accident. As a result he had three cases of post-operative embolism within three months. As Dr. Cazin pointed out, the *lever précoce* will not prevent thrombosis, nor does the danger lie in the thrombosis, but in the clot that becomes detached. It is quite certain that any movement favours such a detachment, and it is impossible to determine beforehand those cases in which thrombosis and embolism are likely to take place.

---

In an article entitled "Auto-protection of the Organism by the Lipoids," Professors Gérard and Lemoine seek to demonstrate that a large number of the protecting or antitoxic bodies are of the nature of lipoids, and are probably in a large measure manufactured in the liver. Phisalix has shown that cholesterin, at one time supposed to be merely an excremental product, is able to neutralise the toxic action of cobra venom, and it has been further found to be antihæmolytic. The authors have also found other lipoids in the liver and bile with still more marked antitoxic powers, among them especially oxycholesterin, ether oxide of cholesterin. These lipoids are characterised by their solubility in ether, benzine, and chloroform. They are insoluble in water and form colloidal solutions in the presence of certain phosphorus compounds termed phosphatides. Their antitoxic properties are probably attributable to this colloidal state. It is pointed out that these lipoids are diffused throughout the organism, but they are especially prominent in those parts most exposed to infection, and where, therefore, their antitoxic powers are most in requisition, such as the red and white corpuscles of the blood and the lungs. The nervous system also, where protection against infection is of such primary importance to the organism, is especially rich in these substances. On the other hand, the skeleton contains little or none. Moreover, according to these authors, in an infected organ these antitoxic lipoids increase in quantity, and are transferred hither from other parts of the economy, and they suggest that the so-called fatty degeneration of infected areas is really the result of attempted protection on the part of the organism against the infective processes.

Dr. H. Bordier, of Lyons, has published some successful results obtained in the treatment of uterine fibroids by the Röntgen rays. Previous attempts have been more or less unsatisfactory, owing to the fact that doses of sufficiently penetrating rays were liable to set up a dermatitis. Now by the use of ray filters in the form of aluminium sheets, the superficial effects are avoided, and rays of a deep penetration can be used for the purpose. Dr. Bordier uses aluminium sheets of 0.5 to 1.5 millimetres thickness. He makes about six applications between the periods. The dose of the rays is regulated by means of the chromo-radiometer, so as to avoid all risk of a radio-dermatitis. Five cases have been treated, two of which are still under treatment, although they may be considered cured, as the fibromata and periods have disappeared. The other three cases have remained cured for several months. The effect is explained in a certain measure by the fact that the rays exert a destructive action upon the embryonic cells of the fibroma, and so cause a progressive atrophy of the tumour, but the author thinks that an important factor in the cure is the production of an artificial menopause. It has been shown that the rays have an elective action upon the ovaries, and may cause an atrophy of these glands. This disappearance of the periods is accompanied by the same general symptoms (flushings, etc.), as in the natural menopause, but no ill-effects have been observed, such as follow sometimes the removal of the ovaries. Possibly the ovary, although atrophied, still retains its power of internal secretion for some time, so that the menopause does not occur in the same sudden manner as with extirpation of the ovaries. In any case Dr. Bordier has not observed any untoward symptom as a result of this artificial menopause, although in two cases the periods have ceased for over six months.

---

Various and opposite opinions have been expressed as to the efficacy of the calcium salts in the treatment of Bright's disease. Excellent results have been claimed for the treatment by Dr. Iscovesco, and more recently Dr. P. Tumminia, of Palermo, has published a report upon the treatment in 20 cases admitted to the hospital there, in which he also obtained very satisfactory results. He administered calcium chloride in doses of 0.5 to 1 gramme per day, and in order to test properly the effects of the drug he first put the patients on a purely milk diet for ten days, during which time the urine was estimated each day and analysed chemically and microscopically. Then, for a further period of ten days, the same diet was continued, and the calcium chloride administered as mentioned above, the daily analyses of urine being continued as before. The calcium chloride was then discontinued again, the analysis being made each day for a further ten days. The effect of the calcium chloride on the blood-pressure was also observed by measuring the blood-pressure before, during, and after the treatment. In three cases the effect was quite beyond expectation. The daily quantity of urine rapidly increased and attained the normal; the specific gravity increased, and the albuminuria and casts disappeared. The blood-pressure was also notably raised. In thirteen other cases the condition improved considerably under the treatment, but the effect was not so marked. There was increase in quantity and specific gravity and diminution of albumen and casts, but the albumen and casts did not altogether disappear. In the four remaining cases the ten days' treatment with calcium chloride appeared to produce no good results. It would appear, therefore, from these observations that calcium chloride is a useful medicament in certain cases of Bright's disease.

---

No longer ago than in our issue of August 28, 1909 (p. 563) attention was directed to the accounts given in the United States of the occurrence and progress of Pellagra in that continent, and the possibility of the outbreak of the disease in Britain was remarked upon. In the September number of the *Edinburgh Medical Journal* Drs. Brown and Low report on a Shetland woman who has been under their care in the Royal Edinburgh Asylum, suffering from the disease; this is believed to be only the second case on record in the kingdom. The patient first felt out of sorts in July 1907, but continued her occupation of gutting and salting fish until the following spring, when she complained of weakness in the knees and nasal catarrh, with amenorrhœa. In May 1908 she had difficulty in walking and diarrhœa, and a month later mental excitement with mild delirium set in, lasting five days. A little later delusions and hallucinations supervened, and she was admitted to the Asylum. She was thin and anæmic, unable to walk or stand, and there was well-marked spasticity of both legs; severe stomatitis and a septic vaginal discharge were present. An eruption extended all over the forehead, cheeks, nose, chin, ears, and upper part of the neck; wherever, in fact, there had been exposure to light. It resembled a seborrhœic dermatitis with added pigmentation. Pigmented also were the backs of the fingers, hands, wrists, and lower forearms, and the dorsa of the toes. These eruptions are described as typically those of pellagra. The patient got gradually worse and died; no post-mortem examination was permitted. The woman had never eaten maize, but bread made from barley instead of wheat is sometimes consumed in Shetland.

---

The treatment of Buboes by injection of iodoform in vaseline is advocated in the *Journal de Med. et de Chir. Prat.* by Tartavez as superior to the usual plans of free incision and drainage. This author recommends that, after careful sterilisation of the skin, a puncture be made into the bubonic cavity with a fine bistoury; the purulent contents are then expressed or curetted away, a proceeding which may necessitate the preliminary injection of cocaine. After thus getting rid of the pus the cavity is flushed out with a stream of oxygenated water, and then the medicament is injected from an ordinary urethral syringe. The mixture consists of one part of iodoform in ten parts of vaseline, heated to a temperature of 45° C. to ensure complete liquefaction. The injection is to be carried out slowly, and the cavity must be filled, but not overdistended. Should the abscess be a large one the proportion of iodoform is reduced to half the above, owing to the risk of iodoform poisoning. A dressing of iodoform gauze and collodion is fixed over the site of puncture, and the patient remains at rest in bed. Pain disappears in a few hours, and the dressings are not changed for four days, by which time a cure has usually been effected.

---

A very elaborate series of animal experiments into the Toxic Action of Chloroform upon the various organs of the body, but especially the liver, is reported by Drs. Whipple and Sperry in the *Johns Hopkins Hospital Bulletin.* It would appear that chloroform produces certain necrotic and degenerative changes in the hepatic lobules with extraordinary constancy, whereas ether apparently does not. From their summary of conclusions the following *dicta* are selected. Chloroform anæsthesia for a period of an hour or upwards always causes some central liver necrosis, both in man and animals. Animals vary widely in susceptibility to this drug, and young animals are more susceptible than adults. Chloroform anæsthesia for as little as 35 minutes has been followed in man by delayed chloroform poisoning with almost complete liver necrosis. The essential pathological change in both dogs and man is extensive necrosis and fatty degeneration of the liver, and there may also be ecchymoses and hæmorrhages into the peritoneum or upper intestinal tract. Pregnancy is no protection against chloroform toxæmia. The necrosis begins centrally in the lobule, and is uninfluenced by the blood supply; it becomes visible to the microscope only some six to ten hours after anæsthesia. Repair takes place rapidly, and the normal appearances of the cells are regained in two to three weeks; it is not followed by cirrhosis, and it goes on just the same when arterial blood is excluded from the liver.

---

The enormous size which Ovarian Cysts may attain, and the rapidity of their growth, are well illustrated by the record of one operated upon by Dr. S. H. Knight, published in the *American Journal of Obstetrics and Gynæcology.* This case is worthy of a place beside the classical account of a similar cyst in France, details of which are given in Mr. Bland-Sutton's book on Tumours. The patient in question was by nationality a Hungarian, and she had noticed the presence of a mass within her abdomen for ten months; she had had one child. At the operation it was found that the tumour was a multilocular adenoma; clear ovarian fluid escaped in large quantities from a trocar, but some of the daughter cysts contained a thin pea-soup-like fluid. The solid part of the growth weighed 15 pounds, the liquid collected amounted to 96 pints: the whole mass thus easily outweighed the rest of the patient, for when she left hospital after a speedy and uneventful convalescence, her weight was ascertained to be 87 pounds only. The photograph with which the article is illustrated gives a vivid picture of the enormous abdominal distension from which the patient suffered.

---

An elaborate review of the cervical or suprapubic Cæsarean section advocated and introduced three years ago by Frank has been read to the Chicago Gynæcological Society by Dr. Lewis. This author has collected from the German, Austrian and Russian literature 102 cases of the operation, which was designed by its originator especially to meet those cases in which the probability of septic infection of the uterus, owing to long rupture of the membranes or examination by the uncleanly, renders the Posso operation particularly dangerous. The principle followed has been to make a transverse abdominal incision not far above the pubes. Strip up the vesico-uterine pouch of peritoneum and open the uterus by a transverse cut through the circular muscle fibres of its lower segment. The uterus is then sewn up, after delivery of the child, with or without drainage into the vagina. In later modifications the peritoneum is opened and then closed again by permanent or temporary sutures before the uterus is opened. Lewis asks, Does cervical Cæsarean section deserve a place in obstetric therapeutics? Is it better adapted for infected cases, or probably infected cases, than other means at our command? He concludes after a survey of the reported cases that the results of the operation are not as brilliant as its advocates maintain. He points out that it is not difficult to wall off the general peritoneal cavity by properly placed laparotomy pads from any contact with uterine contents, and he also insists that connective tissue spaces such as those opened up by the cervical section are far less able to deal successfully with bacteria than is the peritoneum itself. He says the operation is more difficult and takes longer than the old one, involves considerable difficulty in the extraction of the child, and leaves the uterus in an abnormal position. For clean cases it cannot replace the ordinary method; for cases of possible previous infection it offers no better chances than the classical operation with careful damming of the peritoneal cavity against the contents of the uterus and tight suturing of the uterine walls, while for cases of certain infection it cannot replace craniotomy.

---

Vaginal Cæsarean section, on the other hand, is strongly recommended in the same issue of the *American Journal of Obstetrics and Gynæcology* by Dr. W. M. Sprigg, of Washington. The indications for this operation are given as two in number—when the life of mother or child is in grave danger, and the uterus must be quickly emptied through a rigid and unyielding cervix; and when manual or instrumental delivery is certain to be attended with lacerations, as in carcinoma of the cervix, stenosis, rigidity from scar tissue, and the like. In determining the need for vaginal section the question of time is all important; a cervix that can be dilated in an hour or two cannot be considered very rigid, but if the mother is in eclampsia it may not be possible to wait so long. This latter condition, as a matter of fact, accounts for far the largest number of cases in which the operation is indicated; it is claimed that manual dilatation adds distinct risks of subsequent sepsis to the dangers the eclamptic patient already undergoes, and that by performing vaginal Cæsarean section the vagina can be rendered and maintained perfectly aseptic. Cervical incision is of less value because of the length of time still required to dilate the lower uterine segment. Certain cases of placenta prævia are also described as suitable for vaginal Cæsarean section, which is performed as follows: The patient is put in the lithotomy position, the perineum depressed with a retractor, and the cervix seized in a tenaculum or held with traction sutures. A sound is passed into the bladder to locate the lowest point of its attachment to the uterus. The lateral fornices are then opened, and the bladder is pushed off the uterus by blunt dissection to a point about four inches up; a long retractor then keeps the bladder out of the way and exposes the uterus to the highest point of the intended incision. With straight, blunt scissors the cervix and uterus are incised in the median line far enough up to admit of lesion or the application of forceps. The membranes, which have been protected by a finger of the left hand, then prolapse into the wound, and can be ruptured; the fœtus is delivered by forceps or version, the placenta and membranes extracted, and the uterus washed out with hot saline solution. If there is much bleeding this should be controlled with gauze packing. The median incision is then sutured, and the vagina loosely packed with gauze.

---

The advantages of morphine with atropine, or of the scopolamine-morphine combination, in facilitating delivery and robbing labour of many of its terrors have been much praised of late years, chiefly on the Continent. The same subject was briefly discussed in these columns a year ago (The Hospital, November 21, 1908, p. 187); but the literature on the subject in this country is still scanty. In Canada Drs. Halpenny and Vrooman have given the scopolamine-morphine method an extensive trial, and are highly pleased with it. Several observers in Germany who have reported large series of cases have noted certain disadvantages, such as a mild delirium occasionally, which seems to have no other ill-effect than the necessity for excluding the relatives and friends, and a tendency to post-partum hæmorrhage. But these two Winnipeg physicians do not believe that the latter phenomenon has anything to do with the administration, and the former has never in their experience been of any consequence. They find that when carefully administered these drugs will safely alleviate consciousness of the pains of labour, and in many cases abolish any remembrance of them. They think delivery is hastened rather than delayed, though this they are not prepared to prove mathematically. They are satisfied that the child suffers no ill effects; but, owing to the necessity for absolute quiet after the hypodermic injection is given, they recommend its use only when the patient is in hospital or attended by a good trained nurse.

